# Supplementary material for: Combined population and patient-specific brain networks predict electrical stimulation propagation
Source: medRxiv. 2026 Oct 1:2026.09.29.26364360. Preprint. [Version 1] doi: 10.64898/2026.09.29.26364360 (PMC13648782; doi:10.64898/2026.09.29.26364360)
Supplement: Supplementary file 1 [file NIHPP2026.09.29.26364360V1-supplement-1.pdf]

medRxiv preprint doi: https://doi.org/10.64898/2026.09.29.26364360; this version posted October 1, 2026. The copyright holder for this preprint (which was not certified by peer review) is the author/funder, who has granted medRxiv a license to display the preprint in perpetuity.
It is made available under a CC-BY 4.0 International license .

SUPPLEMENTAL MATERIAL

SUPPLEMENTARY METHODS

SM1. CCEP acquisition hardware and artifact rejection

Depth electrodes had 6–12 cylindrical contacts (2.41 mm length, 1.1 mm diameter, 5 mm inter-contact spacing). Single-pulse stimulation was delivered with a Nicolet Cortical Stimulator (Natus) across all or a subset of adjacent bipolar pairs. iEEG was sampled at 512-1024 Hz. Trials with extreme voltages (> 1000 μV) were excluded, and additional artifact rejection was applied to the averaged waveforms. CCEP detections were reviewed by a board-certified epileptologist, and data were retained using a positive predictive value (PPV) threshold >0.7. Channels with high impedance, outside the brain, or in dural proximity were excluded from stimulation, and trials producing a stimulation-induced seizure were removed[34].

SM2. Electrode localization mechanics

Contacts were localized with iEEG-recon[48]: post-implant CT was rigidly registered (6 DOF) to pre-implant T1 MRI, contacts were semi-automatically identified in VoxTool, and each contact was assigned to the Lausanne 2008 (scale 33; 82 regions) ROI of highest volumetric overlap with a 2 mm-radius sphere centered at the contact. Only gray-matter contacts were retained. A pair was labeled responsive if any trial between its two ROIs evoked a significant N1.

SM3. Quantifying SOZ extent (number of SOZ electrodes)

From the clinically determined seizure-onset-zone electrode list for each patient, we computed the number of SOZ electrodes, setting patients with no recorded seizures to zero and excluding patients without an SOZ label (n = 33 with usable labels). This count indexes the spatial extent of the epileptogenic zone and is used in Figure S5 (its distribution by SOZ and implant laterality) and Figure S6 (against patient AUC).

SUPPLEMENTARY RESULTS

SR1. Model predictions are robust to clinical variation *(Figures S4, S5; Table S5)*

The relationships between population and patient-specific networks described by our models have translational potential for guiding stimulation therapy, which would require performance that is robust across the heterogeneity of clinical practice. We therefore tested whether patient-level model performance could be explained by clinical or implant-strategy variables. Although performance varied across patients (full-model AUC 0.84 [0.79, 0.87]), it was not associated with any continuous covariate (Figure S4), mean Euclidean distance ($r = 0.13$, $p = 0.48$), number of stimulation-response pairs ($r = -0.15$, $p = 0.40$), connection rate ($r = 0.02$, $p = 0.93$), response rate ($r = -0.26$, $p = 0.14$), or long- and short-range pair rates ($|r| = 0.19$, $p = 0.28$), indicating that performance is not driven by implant density or sampling. Likewise, AUC did not differ by sex ($p = 0.92$), surgical intervention (all Bonferroni $p = 1.0$), implant laterality (all Bonferroni $p > 0.6$), or imaging findings on MRI (potential epileptogenic lesion vs. nonspecific

medRxiv preprint doi: https://doi.org/10.64898/2026.09.29.26364360; this version posted October 1, 2026. The copyright holder for this preprint (which was not certified by peer review) is the author/funder, who has granted medRxiv a license to display the preprint in perpetuity.
It is made available under a CC-BY 4.0 International license .

abnormality vs. normal; all Bonferroni p = 1.0; Figure S5). Model performance is therefore stable across the clinical variation of the cohort, supporting its potential generalization to new patients.

SR2. Performance is not associated with SOZ lateralization or extent *(Figures S6, S7; Table S5)*

We next tested whether performance varied with the epileptogenic network itself, using the curated SOZ labels now available for 30 of the 34 patients (Figure S5). No epileptogenic-network variable was significantly associated with AUC after correction. Right-lateralized SOZs had numerically higher AUC than left-lateralized SOZs (median 0.87 [0.86, 0.88] vs. 0.83 [0.78, 0.86]), but this difference did not survive multiple-comparison correction (Mann-Whitney, raw p = 0.032, Bonferroni p = 0.097), and neither bilateral SOZs nor SOZ region/localization differed (all Bonferroni p > 0.2). Performance was likewise independent of the *extent* of the epileptogenic zone: the number of clinically determined SOZ electrodes did not correlate with patient AUC (r = -0.03, p = 0.88, n = 33; Figure S6). Descriptively, SOZ-electrode counts tracked laterality and implant breadth rather than performance—patients with bilateral SOZs carried more SOZ electrodes (median 12.5) than left- (5.5) or right-lateralized (7.0) patients, and contralateral-skewed implants more than unilateral ones (Figure S5). Together with SR1, model performance was not explained by any clinical, implant, or epileptogenic-network characteristic of the cohort. (The cohort still contains more left- than right-lateralized SOZs, 16 vs. 8, consistent with greater clinical detection of left-hemisphere seizures; the residual numerical right > left trend should be read in that light.)

SUPPLEMENTARY FIGURES

medRxiv preprint doi: https://doi.org/10.64898/2026.09.29.26364360; this version posted October 1, 2026. The copyright holder for this preprint (which was not certified by peer review) is the author/funder, who has granted medRxiv a license to display the preprint in perpetuity.
It is made available under a CC-BY 4.0 International license .

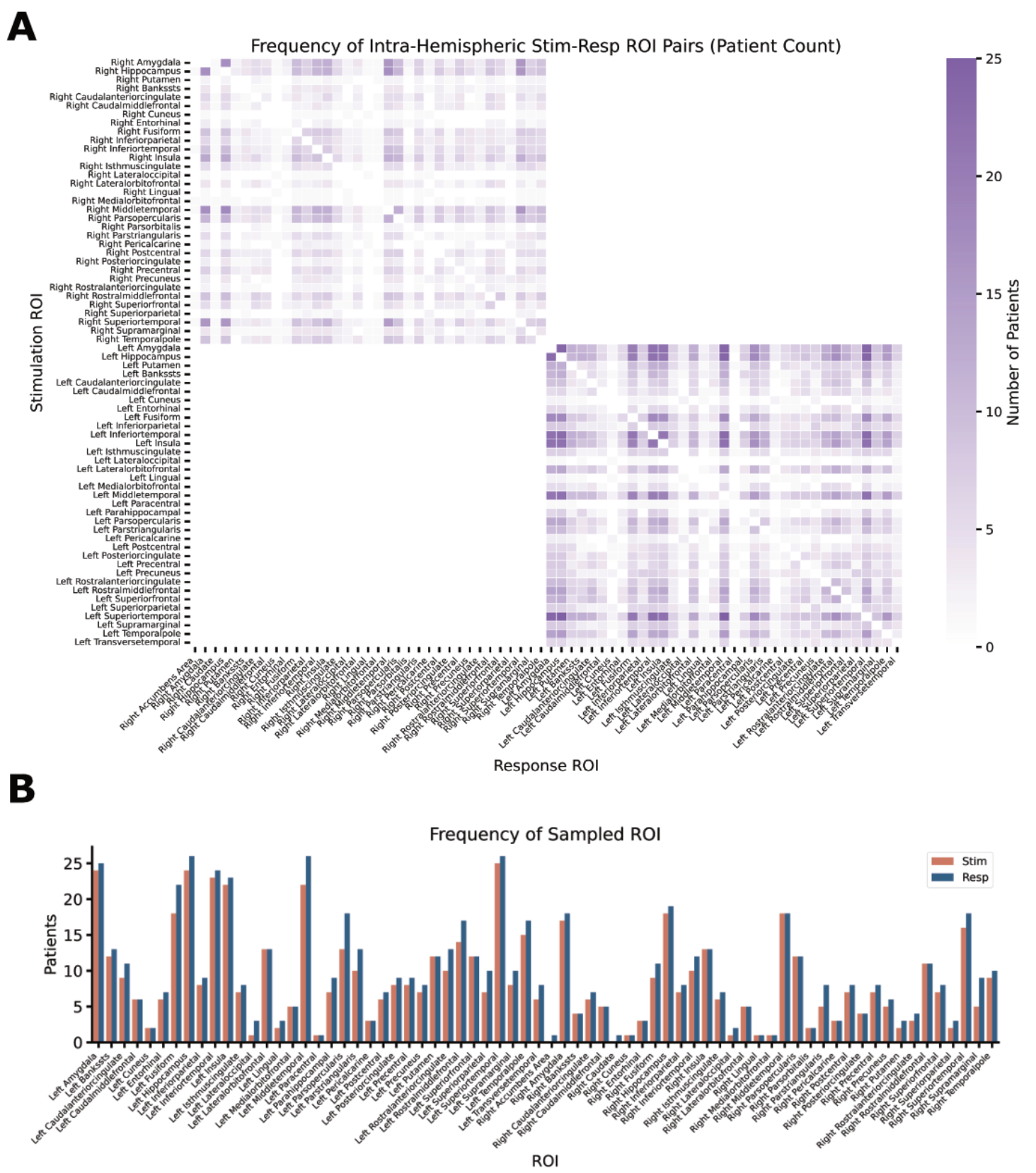

Figure S1 : Frequency of Intra-Hemispheric stimulation-response ROI pairs in the HUP dataset. (A) The number of samples within a specific stimulation and response pair across the HUP dataset at the ROI level according to DK atlas. The rows are stimulation ROIs, the columns are response ROIs and the elements are the number of patients. **(B)** Number of patients with samples in specific ROIs, stratified by stimulation and response.

medRxiv preprint doi: https://doi.org/10.64898/2026.09.29.26364360; this version posted October 1, 2026. The copyright holder for this preprint
(which was not certified by peer review) is the author/funder, who has granted medRxiv a license to display the preprint in perpetuity.
It is made available under a CC-BY 4.0 International license .

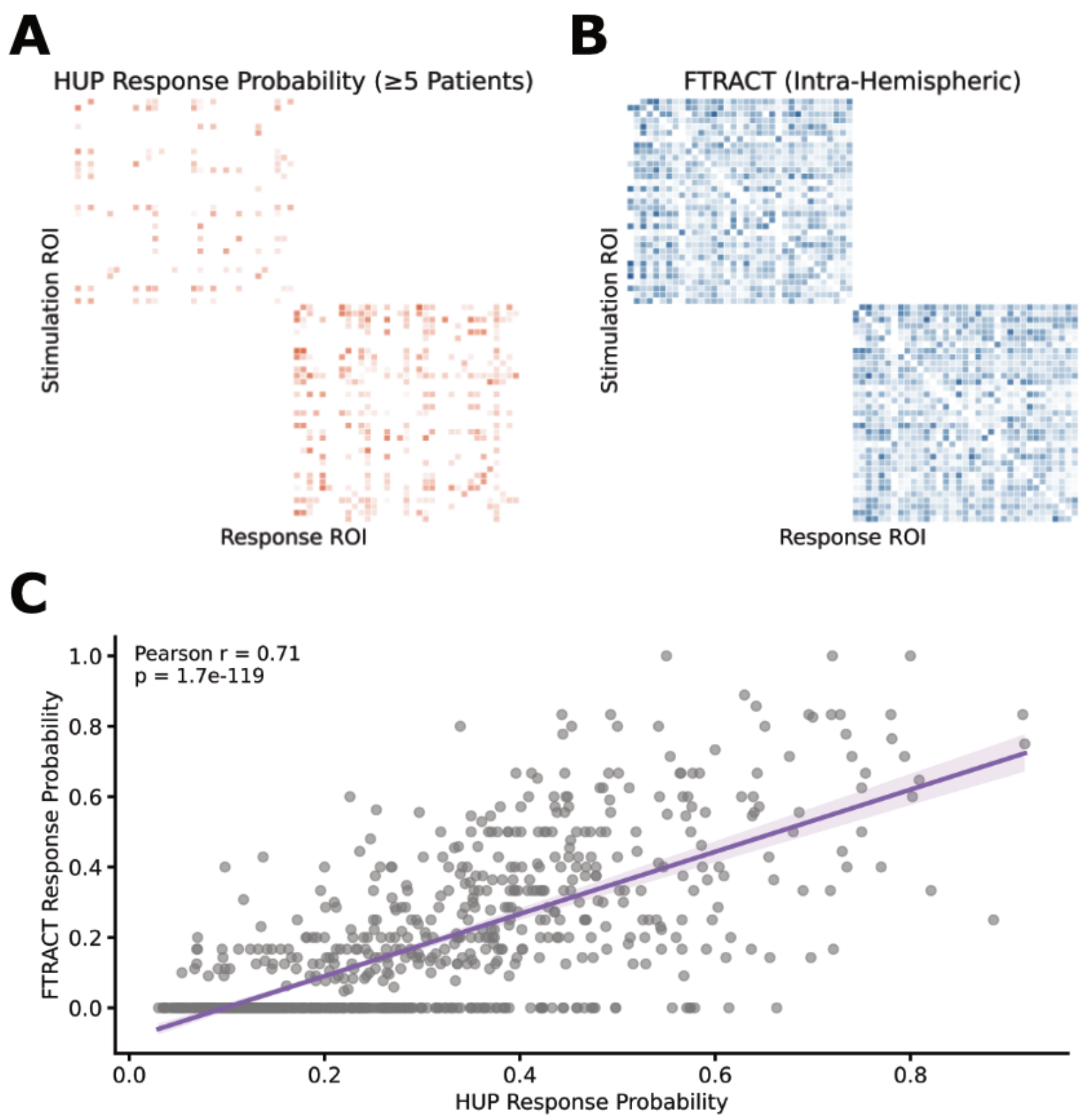

Figure S2 : Correlation between F-TRACT response probabilities and HUP response probabilities

medRxiv preprint doi: https://doi.org/10.64898/2026.09.29.26364360; this version posted October 1, 2026. The copyright holder for this preprint (which was not certified by peer review) is the author/funder, who has granted medRxiv a license to display the preprint in perpetuity.
It is made available under a CC-BY 4.0 International license .

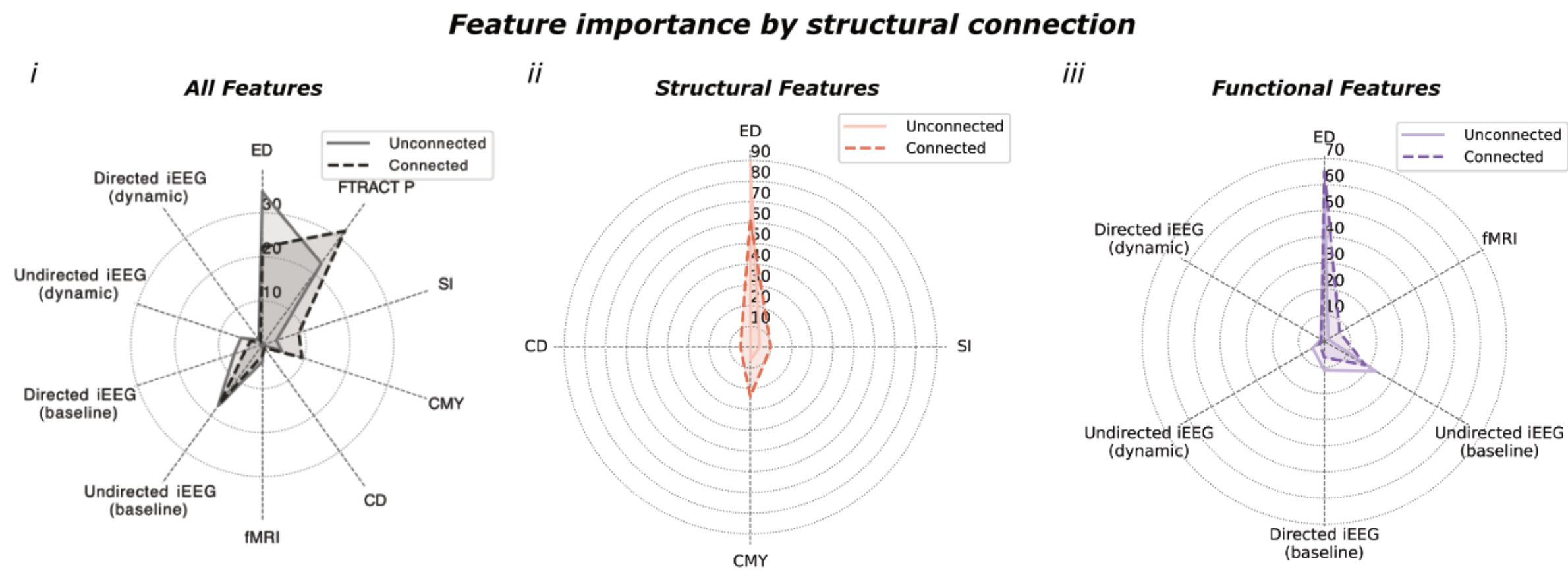

Figure S3: Comparison of feature importance between model with structurally connected vs unconnected stimulation-response pairs.

medRxiv preprint doi: https://doi.org/10.64898/2026.09.29.26364360; this version posted October 1, 2026. The copyright holder for this preprint (which was not certified by peer review) is the author/funder, who has granted medRxiv a license to display the preprint in perpetuity.
It is made available under a CC-BY 4.0 International license .

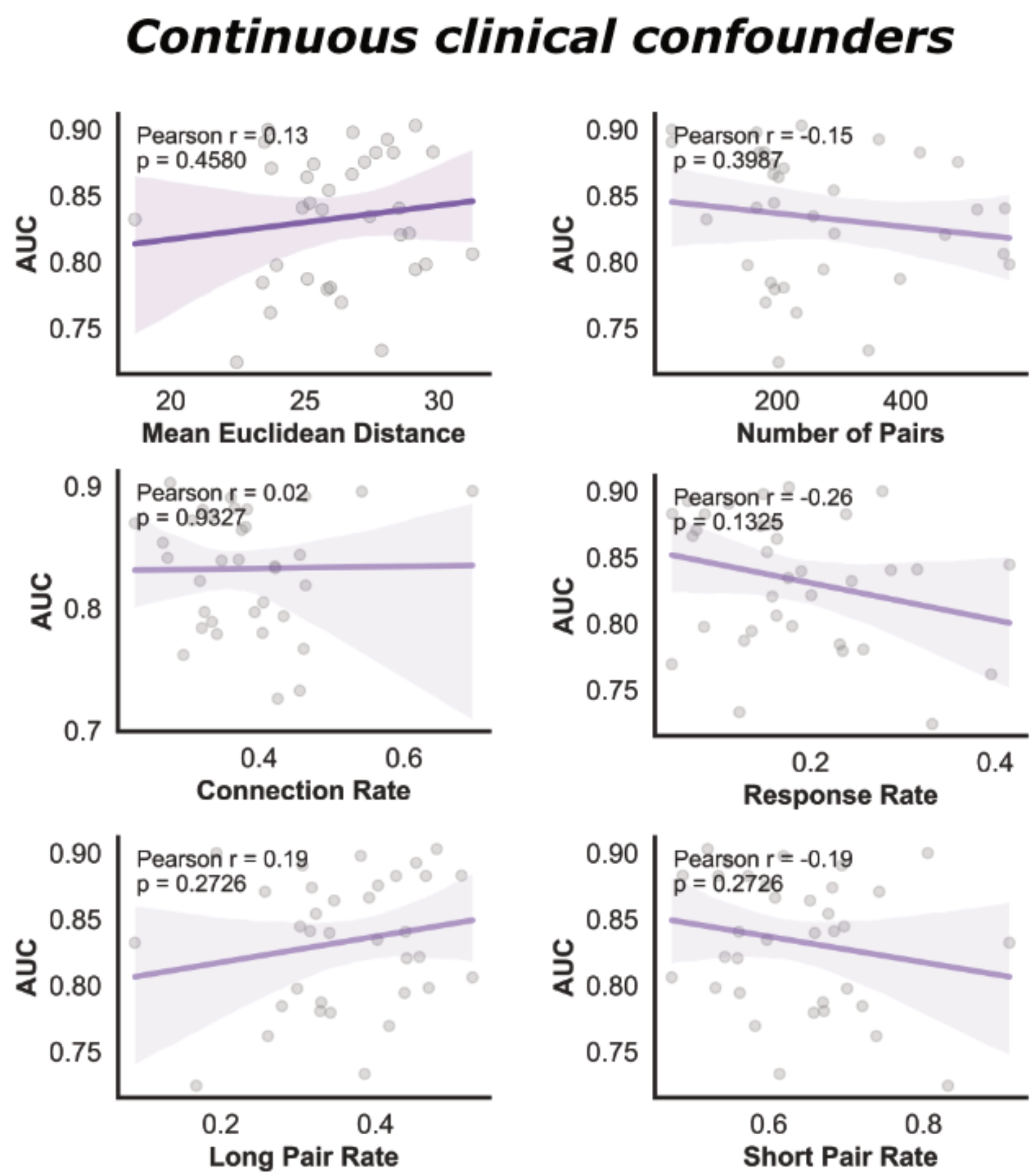

Figure S4: Correlation between patient-level AUCs and continuous clinical confounders

medRxiv preprint doi: https://doi.org/10.64898/2026.09.29.26364360; this version posted October 1, 2026. The copyright holder for this preprint (which was not certified by peer review) is the author/funder, who has granted medRxiv a license to display the preprint in perpetuity.
It is made available under a CC-BY 4.0 International license .

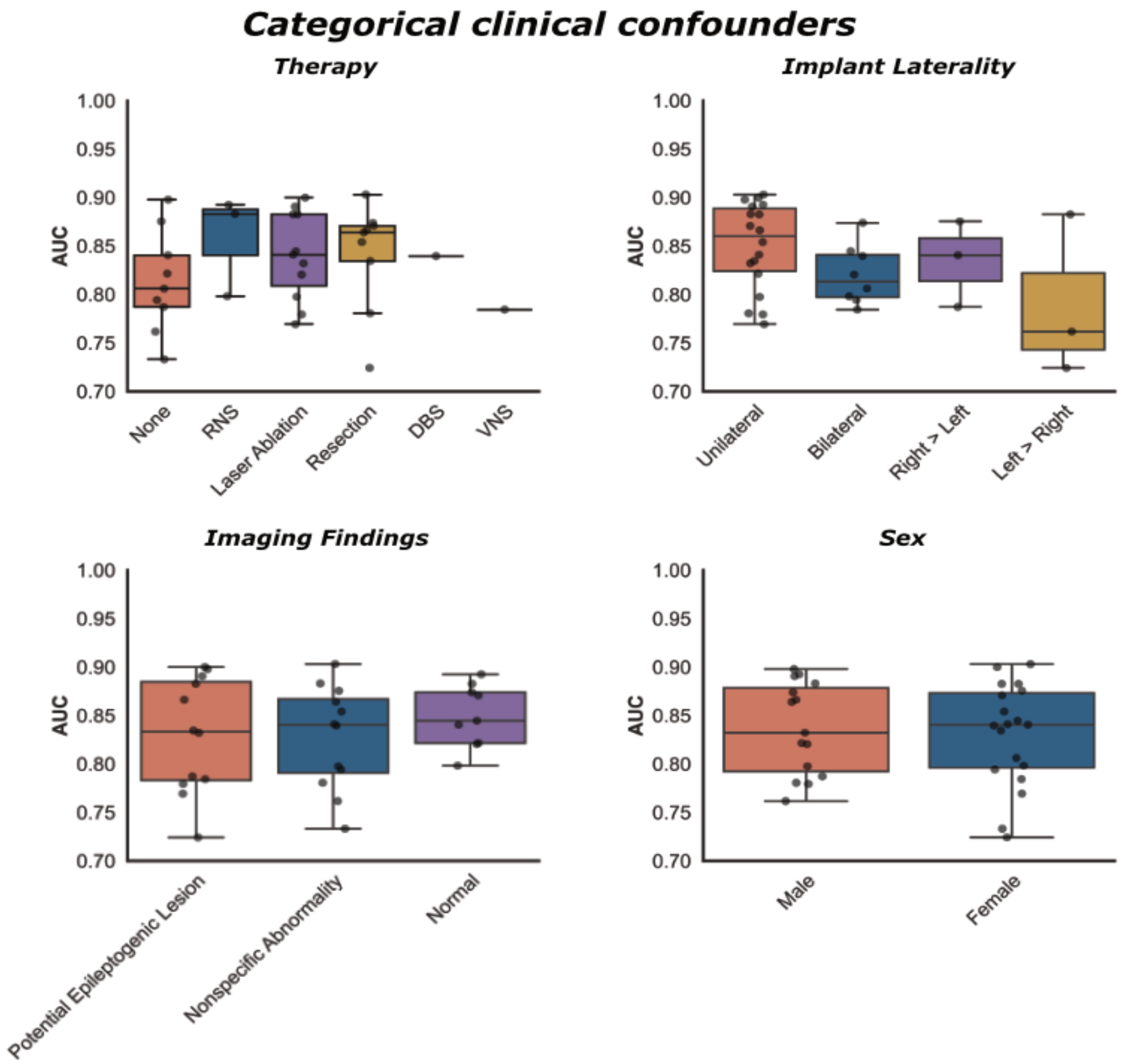

Figure S5: Comparison between patient-level AUCs and categorical clinical confounders.

medRxiv preprint doi: https://doi.org/10.64898/2026.09.29.26364360; this version posted October 1, 2026. The copyright holder for this preprint
(which was not certified by peer review) is the author/funder, who has granted medRxiv a license to display the preprint in perpetuity.
It is made available under a CC-BY 4.0 International license .

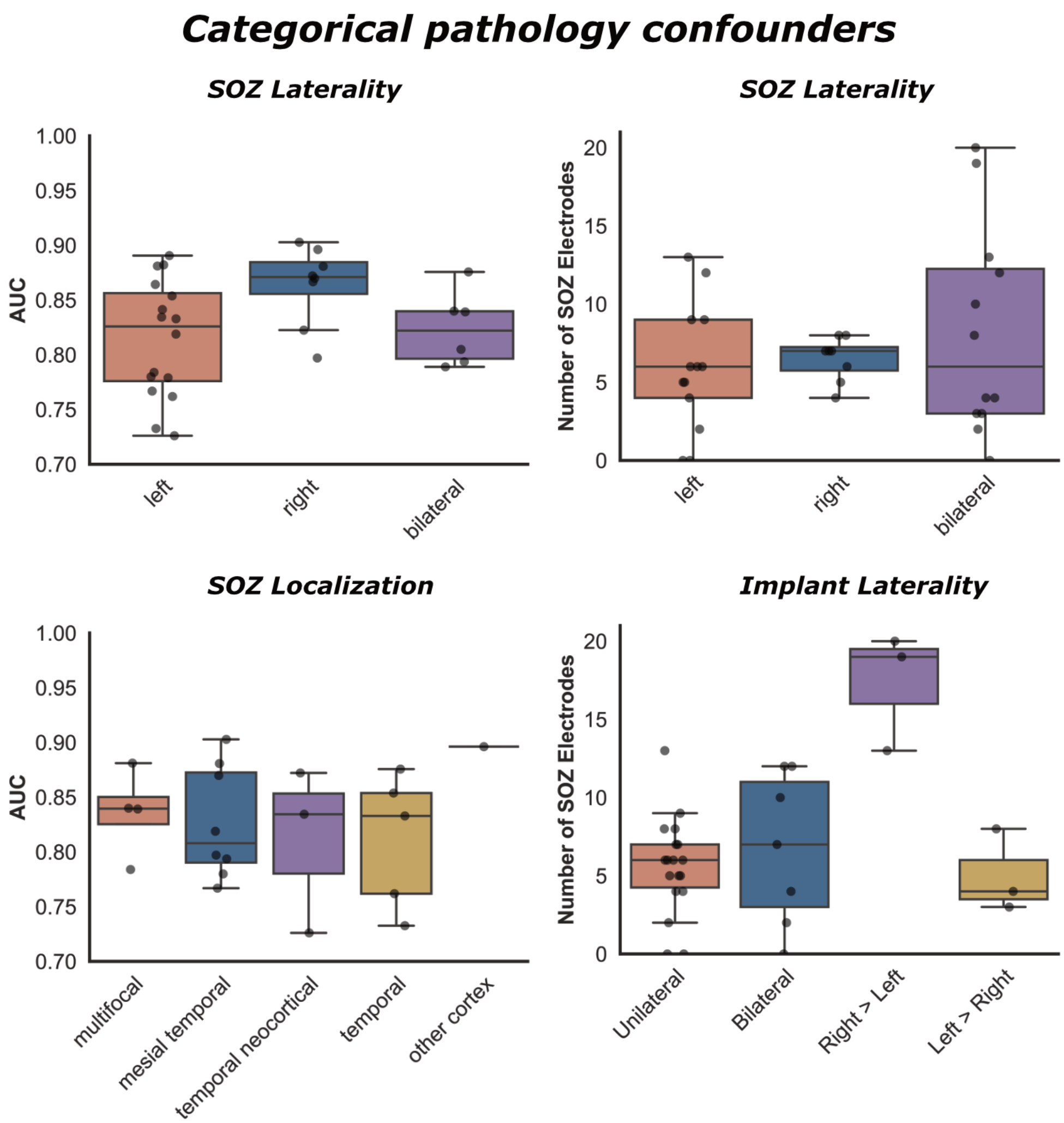

Figure S6: Comparison between patient-level AUCs and categorical pathology confounders.

medRxiv preprint doi: https://doi.org/10.64898/2026.09.29.26364360; this version posted October 1, 2026. The copyright holder for this preprint (which was not certified by peer review) is the author/funder, who has granted medRxiv a license to display the preprint in perpetuity.
It is made available under a CC-BY 4.0 International license .

SUPPLEMENTARY TABLES

Table S1 : Correlation between features and binary N1 response

medRxiv preprint doi: https://doi.org/10.64898/2026.09.29.26364360; this version posted October 1, 2026. The copyright holder for this preprint (which was not certified by peer review) is the author/funder, who has granted medRxiv a license to display the preprint in perpetuity.
It is made available under a CC-BY 4.0 International license .

Panel	Condition	Feature	|ρ| [95% CI]	Per-patient r, median (signed)	n (patients)	p (FDR)
A. Control condition (all pairs)	Unadjusted	Euclidean distance	0.36 [0.35, 0.38]	-0.37	34	<0.001
A. Control condition (all pairs)	Unadjusted	F-TRACT response probability	0.41 [0.39, 0.43]	0.42	34	<0.001
A. Control condition (all pairs)	Unadjusted	Connection density	0.13 [0.10, 0.15]	0.11	34	<0.001
A. Control condition (all pairs)	Unadjusted	Communicability	0.16 [0.14, 0.19]	0.15	34	<0.001
A. Control condition (all pairs)	Unadjusted	Search information	0.08 [0.06, 0.10]	-0.09	34	<0.001
A. Control condition (all pairs)	Unadjusted	Undirected FC, baseline	0.29 [0.27, 0.32]	0.28	34	<0.001
A. Control condition (all pairs)	Unadjusted	Undirected FC, dynamic	0.27 [0.25, 0.30]	0.27	34	<0.001
A. Control condition (all pairs)	Unadjusted	Directed FC, baseline	0.00 [0.00, 0.02]	-0.01	34	0.919
A. Control condition (all pairs)	Unadjusted	Directed FC, dynamic	0.02 [0.00, 0.04]	-0.01	34	0.508
A. Control condition (all pairs)	Unadjusted	fMRI functional connectivity	0.03 [0.01, 0.05]	0.06	34	0.005
A. Control condition (all pairs)	Distance-adjusted	Euclidean distance	0.36 [0.35, 0.38]	-0.37	34	<0.001
A. Control condition (all pairs)	Distance-adjusted	F-TRACT response probability	0.23 [0.21, 0.25]	0.23	34	<0.001
A. Control condition (all pairs)	Distance-adjusted	Connection density	0.08 [0.05, 0.10]	0.06	34	0.022
A. Control condition (all pairs)	Distance-adjusted	Communicability	0.10 [0.07, 0.12]	0.07	34	<0.001
A. Control condition (all pairs)	Distance-adjusted	Search information	0.02 [0.00, 0.04]	-0.04	34	0.034
A. Control condition (all pairs)	Distance-adjusted	Undirected FC, baseline	0.17 [0.15, 0.20]	0.18	34	<0.001
A. Control condition (all pairs)	Distance-adjusted	Undirected FC, dynamic	0.16 [0.13, 0.18]	0.16	34	<0.001
A. Control condition (all pairs)	Distance-adjusted	Directed FC, baseline	0.02 [0.00, 0.04]	-0.02	34	0.04

medRxiv preprint doi: https://doi.org/10.64898/2026.09.29.26364360; this version posted October 1, 2026. The copyright holder for this preprint (which was not certified by peer review) is the author/funder, who has granted medRxiv a license to display the preprint in perpetuity.
It is made available under a CC-BY 4.0 International license .

A. Control condition (all pairs)	Distance-adjusted	Directed FC, dynamic	0.00 [0.00, 0.02]	-0.04	34	0.025
A. Control condition (all pairs)	Distance-adjusted	fMRI functional connectivity	0.01 [0.00, 0.03]	0.01	34	0.134
A. Control condition (all pairs)	F-TRACT-adjusted	Euclidean distance	0.12 [0.10, 0.14]	-0.12	34	<0.001
A. Control condition (all pairs)	F-TRACT-adjusted	F-TRACT response probability	0.41 [0.39, 0.43]	0.42	34	<0.001
A. Control condition (all pairs)	F-TRACT-adjusted	Connection density	0.02 [0.00, 0.05]	0	34	0.904
A. Control condition (all pairs)	F-TRACT-adjusted	Communicability	0.03 [0.00, 0.05]	0.01	34	0.904
A. Control condition (all pairs)	F-TRACT-adjusted	Search information	0.02 [0.00, 0.04]	0	34	0.904
A. Control condition (all pairs)	F-TRACT-adjusted	Undirected FC, baseline	0.17 [0.14, 0.20]	0.15	34	<0.001
A. Control condition (all pairs)	F-TRACT-adjusted	Undirected FC, dynamic	0.15 [0.13, 0.18]	0.13	34	<0.001
A. Control condition (all pairs)	F-TRACT-adjusted	Directed FC, baseline	0.01 [0.00, 0.03]	0	34	0.946
A. Control condition (all pairs)	F-TRACT-adjusted	Directed FC, dynamic	0.02 [0.00, 0.04]	-0.02	34	0.904
A. Control condition (all pairs)	F-TRACT-adjusted	fMRI functional connectivity	0.01 [0.00, 0.03]	0	34	0.904
B. By interregional distance	Short-range (<30 mm)	Euclidean distance	0.28 [0.26, 0.31]	-0.31	34	<0.001
B. By interregional distance	Short-range (<30 mm)	F-TRACT response probability	0.33 [0.30, 0.35]	0.33	34	<0.001
B. By interregional distance	Short-range (<30 mm)	Connection density	0.11 [0.08, 0.14]	0.1	34	<0.001
B. By interregional distance	Short-range (<30 mm)	Communicability	0.14 [0.11, 0.17]	0.13	34	<0.001
B. By interregional distance	Short-range (<30 mm)	Search information	0.06 [0.03, 0.09]	-0.08	34	<0.001
B. By interregional distance	Short-range (<30 mm)	Undirected FC, baseline	0.24 [0.22, 0.27]	0.23	34	<0.001
B. By interregional distance	Short-range (<30 mm)	Undirected FC, dynamic	0.23 [0.21, 0.26]	0.22	34	<0.001

medRxiv preprint doi: https://doi.org/10.64898/2026.09.29.26364360; this version posted October 1, 2026. The copyright holder for this preprint (which was not certified by peer review) is the author/funder, who has granted medRxiv a license to display the preprint in perpetuity.
It is made available under a CC-BY 4.0 International license .

B. By interregional distance	Short-range (<30 mm)	Directed FC, baseline	0.00 [0.00, 0.03]	0	34	0.761
B. By interregional distance	Short-range (<30 mm)	Directed FC, dynamic	0.01 [0.00, 0.04]	-0.03	34	0.284
B. By interregional distance	Short-range (<30 mm)	fMRI functional connectivity	0.02 [0.00, 0.04]	0.03	34	0.049
B. By interregional distance	Long-range (≥30 mm)	Euclidean distance	0.08 [0.05, 0.10]	-0.1	23	<0.001
B. By interregional distance	Long-range (≥30 mm)	F-TRACT response probability	0.15 [0.11, 0.19]	0.16	23	<0.001
B. By interregional distance	Long-range (≥30 mm)	Connection density	0.09 [0.03, 0.15]	-0.02	23	0.168
B. By interregional distance	Long-range (≥30 mm)	Communicability	0.13 [0.07, 0.19]	0	23	0.1
B. By interregional distance	Long-range (≥30 mm)	Search information	0.05 [0.01, 0.09]	-0.05	23	0.1
B. By interregional distance	Long-range (≥30 mm)	Undirected FC, baseline	0.06 [0.01, 0.11]	0.03	23	0.158
B. By interregional distance	Long-range (≥30 mm)	Undirected FC, dynamic	0.02 [0.00, 0.07]	0.03	23	0.56
B. By interregional distance	Long-range (≥30 mm)	Directed FC, baseline	0.04 [0.01, 0.06]	-0.07	23	0.012
B. By interregional distance	Long-range (≥30 mm)	Directed FC, dynamic	0.02 [0.00, 0.05]	-0.03	23	0.047
B. By interregional distance	Long-range (≥30 mm)	fMRI functional connectivity	0.06 [0.03, 0.09]	0.08	23	0.012
C. By structural connection	Structurally connected	Euclidean distance	0.35 [0.33, 0.38]	-0.34	34	<0.001
C. By structural connection	Structurally connected	F-TRACT response probability	0.41 [0.38, 0.44]	0.41	34	<0.001
C. By structural connection	Structurally connected	Connection density	0.11 [0.08, 0.15]	0.04	34	0.037
C. By structural connection	Structurally connected	Communicability	0.15 [0.11, 0.19]	0.14	34	<0.001
C. By structural connection	Structurally connected	Search information	0.05 [0.01, 0.08]	-0.09	34	0.008

medRxiv preprint doi: https://doi.org/10.64898/2026.09.29.26364360; this version posted October 1, 2026. The copyright holder for this preprint (which was not certified by peer review) is the author/funder, who has granted medRxiv a license to display the preprint in perpetuity.
It is made available under a CC-BY 4.0 International license .

C. By structural connection	Structurally connected	Undirected FC, baseline	0.28 [0.24, 0.31]	0.25	34	<0.001
C. By structural connection	Structurally connected	Undirected FC, dynamic	0.26 [0.23, 0.30]	0.26	34	<0.001
C. By structural connection	Structurally connected	Directed FC, baseline	0.03 [0.00, 0.06]	0.03	34	0.27
C. By structural connection	Structurally connected	Directed FC, dynamic	0.03 [0.00, 0.07]	0.02	34	0.27
C. By structural connection	Structurally connected	fMRI functional connectivity	0.05 [0.02, 0.09]	-0.06	34	0.27
C. By structural connection	Structurally unconnected	Euclidean distance	0.34 [0.32, 0.36]	-0.32	34	<0.001
C. By structural connection	Structurally unconnected	F-TRACT response probability	0.37 [0.34, 0.39]	0.36	34	<0.001
C. By structural connection	Structurally unconnected	Connection density	—	—	—	—
C. By structural connection	Structurally unconnected	Communicability	0.02 [0.00, 0.04]	-0.03	34	0.698
C. By structural connection	Structurally unconnected	Search information	0.01 [0.00, 0.04]	0.02	34	0.573
C. By structural connection	Structurally unconnected	Undirected FC, baseline	0.29 [0.25, 0.32]	0.29	34	<0.001
C. By structural connection	Structurally unconnected	Undirected FC, dynamic	0.26 [0.23, 0.29]	0.26	34	<0.001
C. By structural connection	Structurally unconnected	Directed FC, baseline	0.01 [0.00, 0.04]	-0.03	34	0.329
C. By structural connection	Structurally unconnected	Directed FC, dynamic	0.01 [0.00, 0.04]	-0.05	34	0.152
C. By structural connection	Structurally unconnected	fMRI functional connectivity	0.01 [0.00, 0.04]	0.04	34	0.207

Table S2a: Model performance (AUC)

medRxiv preprint doi: https://doi.org/10.64898/2026.09.29.26364360; this version posted October 1, 2026. The copyright holder for this preprint (which was not certified by peer review) is the author/funder, who has granted medRxiv a license to display the preprint in perpetuity.
It is made available under a CC-BY 4.0 International license .

Model	n (patients)	AUC, median [IQR]	AUPRC, median [IQR]	AUC, mean ± SD
Euclidean distance (ED)	34	0.803 [0.761, 0.847]	0.442 [0.396, 0.532]	0.801 ± 0.059
ED + F-TRACT	34	0.825 [0.786, 0.861]	0.502 [0.418, 0.580]	0.821 ± 0.055
ED + F-TRACT + structural	34	0.828 [0.787, 0.864]	0.504 [0.400, 0.593]	0.820 ± 0.054
ED + F-TRACT + structural + fMRI	34	0.827 [0.788, 0.865]	0.500 [0.401, 0.595]	0.821 ± 0.054
Full model (+ iEEG FC)	34	0.840 [0.794, 0.875]	0.523 [0.431, 0.587]	0.833 ± 0.049

Table S2b: Incremental-model comparisons

Comparison	Δ AUC (median)	p (FDR)	Significant
Euclidean distance (ED) vs ED + F-TRACT	0.022	<0.001	Yes
ED + F-TRACT vs ED + F-TRACT + structural	0.001	0.853	No
ED + F-TRACT + structural vs ED + F-TRACT + structural + fMRI	0	0.853	No
ED + F-TRACT + structural + fMRI vs Full model (+ iEEG FC)	0.012	<0.001	Yes

Table S2c: Predicted response probabilities

True class	n (edges)	Predicted P(response), median [IQR]
No Response	7548	0.27 [0.13, 0.52]
Response	1635	0.69 [0.52, 0.84]

Table S3: Feature importance for full model

medRxiv preprint doi: https://doi.org/10.64898/2026.09.29.26364360; this version posted October 1, 2026. The copyright holder for this preprint (which was not certified by peer review) is the author/funder, who has granted medRxiv a license to display the preprint in perpetuity.
It is made available under a CC-BY 4.0 International license .

Feature	Importance, % (full model)	Per-patient %, median [IQR]	p (FDR vs 0)	Short-range, %	Long-range, %
Euclidean distance	30.6	30.8 [29.5, 31.4]	<0.001	17.7	14.2
F-TRACT response probability	28.6	28.6 [28.0, 29.3]	<0.001	33.8	14.6
Connection density	2	2.0 [1.4, 2.5]	<0.001	3.6	7.4
Communicability	2.9	2.9 [2.4, 3.6]	<0.001	2.6	9.8
Search information	8.1	8.3 [7.5, 8.5]	<0.001	11.3	0.9
Undirected FC, baseline	17.7	17.7 [16.9, 18.5]	<0.001	21	16.2
Undirected FC, dynamic	3.5	3.6 [2.5, 4.4]	<0.001	1.2	15.6
Directed FC, baseline	5.5	5.3 [5.1, 5.9]	<0.001	6.3	10
Directed FC, dynamic	0.6	0.5 [0.2, 0.9]	<0.001	0.7	1.6
fMRI functional connectivity	0.5	0.4 [0.1, 0.7]	<0.001	1.7	9.5

Table S4a: Prediction residuals by SOZ relationship

Stim–response SOZ relationship	n (patients)	|true - predicted|, median [IQR]
nSOZ-nSOZ	30	0.35 [0.33, 0.37]
nSOZ-SOZ	30	0.34 [0.29, 0.37]
SOZ-nSOZ	27	0.35 [0.31, 0.39]
SOZ-SOZ	18	0.48 [0.33, 0.50]

medRxiv preprint doi: https://doi.org/10.64898/2026.09.29.26364360; this version posted October 1, 2026. The copyright holder for this preprint (which was not certified by peer review) is the author/funder, who has granted medRxiv a license to display the preprint in perpetuity.
It is made available under a CC-BY 4.0 International license .

Table S4b:Residual comparisons (SOZ–SOZ vs each)

Comparison	Δ median residual	Effect size r	p (FDR)	Significant
SOZ–SOZ vs nSOZ-nSOZ	0.068	0.43	0.071	No
SOZ–SOZ vs nSOZ-SOZ	0.128	0.53	0.037	Yes
SOZ–SOZ vs SOZ-nSOZ	0.092	0.56	0.037	Yes

Table S4c:Error rates by SOZ relationship

Stim–response SOZ relationship	FPR, pair-level [95% CI]	FNR, pair-level [95% CI]	FPR, patient median	FNR, patient median
nSOZ-nSOZ	0.28 [0.26, 0.29]	0.24 [0.21, 0.26]	0.28	0.21
nSOZ-SOZ	0.27 [0.24, 0.30]	0.12 [0.08, 0.19]	0.29	0.04
SOZ-nSOZ	0.29 [0.26, 0.33]	0.28 [0.21, 0.36]	0.29	0.22
SOZ-SOZ	0.53 [0.41, 0.65]	0.00 [0.00, 0.08]	1	0

Table S4d:Error-rate comparisons (SOZ–SOZ vs each)

Comparison	Metric	Effect size r	p (FDR)	Significant
SOZ–SOZ vs nSOZ-nSOZ	FPR	0.87	0.001	Yes
SOZ–SOZ vs nSOZ-SOZ	FPR	0.87	0.001	Yes
SOZ–SOZ vs SOZ-nSOZ	FPR	0.87	0.001	Yes

medRxiv preprint doi: https://doi.org/10.64898/2026.09.29.26364360; this version posted October 1, 2026. The copyright holder for this preprint (which was not certified by peer review) is the author/funder, who has granted medRxiv a license to display the preprint in perpetuity.
It is made available under a CC-BY 4.0 International license .

SOZ–SOZ vs nSOZ-nSOZ	FNR	0.88	0.002	Yes
SOZ–SOZ vs nSOZ-SOZ	FNR	0.99	0.009	Yes
SOZ–SOZ vs SOZ-nSOZ	FNR	0.96	0.004	Yes

Table S5a: Continuous clinical/implant confounders

**Patient variable**	**n (patients)**	**Pearson r (vs AUC)**	**p**
Mean Euclidean Distance	34	0.13	0.477
Number of Pairs	34	-0.15	0.398
Connection Rate	34	0.02	0.933
Response Rate	34	-0.26	0.138
Long Pair Rate	34	0.19	0.283
Short Pair Rate	34	-0.19	0.283
Number of SOZ Electrodes	33	-0.03	0.884

Table S5b: Categorical clinical confounders (AUC)

**Variable**	**Category**	**n**	**AUC, median [IQR]**	**Pairwise test (Bonferroni)**
Intervention	DBS	1	0.839 [0.839, 0.839]	all n.s.
	Laser Ablation	11	0.841 [0.808, 0.881]	
	No intervention	9	0.805 [0.789, 0.840]	
	RNS	3	0.881 [0.839, 0.886]	
	Resection	9	0.864 [0.834, 0.870]	
	VNS	1	0.784 [0.784, 0.784]	
Implant Lateralization	Bilateral	8	0.812 [0.796, 0.840]	all n.s.
	Left > Right	3	0.762 [0.744, 0.822]	
	Right > Left	3	0.840 [0.814, 0.858]	
	Unilateral	18	0.860 [0.825, 0.888]	

medRxiv preprint doi: https://doi.org/10.64898/2026.09.29.26364360; this version posted October 1, 2026. The copyright holder for this preprint (which was not certified by peer review) is the author/funder, who has granted medRxiv a license to display the preprint in perpetuity.
It is made available under a CC-BY 4.0 International license .

Imaging Findings	Nonspecific Abnormality	12	0.840 [0.790, 0.867]	all n.s.
	Normal	9	0.844 [0.823, 0.872]	
	Potential Epileptogenic Lesion	12	0.834 [0.783, 0.884]	
Sex	Female	19	0.840 [0.795, 0.873]	all n.s.
	Male	15	0.833 [0.793, 0.877]	

Table S5c:AUC by SOZ lateralization / region

Variable	Category	n	AUC, median [IQR]	Pairwise test (Bonferroni)
SOZ Region	Mesial temporal	8	0.808 [0.790, 0.873]	all n.s.
	Multifocal	4	0.840 [0.825, 0.850]	
	Other cortex	1	0.896 [0.896, 0.896]	
	Temporal	5	0.833 [0.762, 0.854]	
	Temporal neocortical	3	0.834 [0.780, 0.853]	
SOZ Lateralization	Bilateral	6	0.822 [0.796, 0.840]	all n.s.
	Left	16	0.826 [0.776, 0.856]	
	Right	8	0.871 [0.856, 0.885]	

Table S5d: SOZ electrode count by laterality

Variable	Category	n	SOZ electrodes, median [IQR]
SOZ Lateralization	Bilateral	6	12.5 [12.0, 17.5]
	Left	16	5.5 [3.8, 8.2]
	Right	8	7.0 [5.8, 7.2]
Implant Lateralization	Bilateral	7	7.0 [3.0, 11.0]
	Left > Right	3	4.0 [3.5, 6.0]
	Right > Left	3	19.0 [16.0, 19.5]
	Unilateral	18	6.0 [4.2, 7.0]
